# New dimensions in acidocalcisome research: the potential of cryo-EM to uncover novel aspects of protozoan parasite physiology

**DOI:** 10.1128/mbio.01662-24

**Published:** 2025-04-08

**Authors:** Ingrid Augusto, Moara Lemos, Wendell Girard-Dias, José de Anchieta Oliveira Filho, Pedro G. Pascutti, Wanderley de Souza, Kildare Miranda

**Affiliations:** 1 Laboratório de Ultraestrutura Celular Hertha Meyer, Centro de Pesquisa em Medicina de Precisão, Instituto de Biofísica Carlos Chagas Filho and Centro Nacional de Biologia Estrutural e Bioimagem, Universidade Federal do Rio de Janeiro, Rio de Janeiro, Brazil; 2 Instituto Nacional de Ciência e Tecnologia em Biologia Estrutural e Bioimagem—Universidade Federal do Rio de Janeiro, Rio de Janeiro, Brazil; 3 Institut Pasteur 27058, Paris, France; 4 Plataforma de Microscopia Eletrônica Rudolf Barth, Instituto Oswaldo Cruz–Fiocruz, Rio de Janeiro, Brazil; 5 Laboratório de Modelagem e Dinâmica Molecular, Instituto de Biofísica Carlos Chagas Filho, Universidade Federal do Rio de Janeiro, Rio de Janeiro, Brazil; 6 Centro Multiusuário para Análise de Fenômenos Biomédicos, Universidade do Estado do Amazonas, Amazonas, Brazil; Instituto Carlos Chagas, Curitiba, Brazil

**Keywords:** *Trypanosoma cruzi*, acidocalcisomes, ion nanodomains, biomolecular condensates, cryo electron microscopy

## Abstract

Cryo-electron microscopy (cryo-EM) has revolutionized structural biology by enabling high-resolution, near-native visualization of macromolecular structures and entire cells. Its application to etiologic agents of diseases is an expanding field, particularly for those caused by viruses or unicellular eukaryotes, such as protozoan parasites and fungi. This review focuses on acidocalcisomes—ion-rich, multifunctional organelles essential for cell physiology and survival in several pathogens. The structure and function of these organelles are examined through a range of electron microscopy techniques, using *Trypanosoma cruzi* as a model. The advantages and limitations of the methods employed to study acidocalcisome morphofunctional organization—such as chemical fixation, plunge and high-pressure freezing, cryo-electron microscopy of vitrified sections (CEMOVIS), freeze-drying, freeze substitution, tomography, and microanalysis using X rays and inelastic scattered electrons—are discussed, alongside their contributions to our current understanding of acidocalcisome structure and function. Recent advances in cryo-EM and its potential to address longstanding questions and fill existing gaps in our understanding of parasite ion mobilization mechanisms and physiology are also discussed.

## INTRODUCTION

Cryo-electron microscopy (cryo-EM) is widely regarded as one of the most transformative innovations in structural biology, revolutionizing the field by enabling the determination of macromolecular structures at near-atomic resolution with unprecedented speed. The detailed structural models generated through cryo-EM have provided invaluable insights into macromolecular function and their roles in biological processes (1). Recognized in 2017 with the Nobel Prize in Chemistry awarded to its developers, cryo-EM has made remarkable progress in recent years, particularly in achieving high-resolution *in situ* studies of whole cells across diverse biological systems (2–5). A compelling example of the impact of cryo-EM in microbiology was its contribution to the development of COVID-19 vaccines, as well as in diagnostics and monitoring immune responses to vaccination (6–9). Notably, cryo-EM remains the only methodology capable of conducting *in situ* analyses in a state closely resembling the native *in vivo* environment, producing highly reliable and biologically relevant results. Nevertheless, despite its significant advancements across various fields of microbiology (10), the application of cryo-EM to studying neglected diseases caused by protozoan parasites remains in its early stages (11, 12).

## CHALLENGES IN APPLYING CRYO-EM TO PROTOZOAN PARASITE RESEARCH

Cryo-electron tomography (cryo-ET) has been applied to a wide range of microorganisms, including protozoan parasites. Like room-temperature EM techniques, one of the main limitations of cryo-ET is the sample thickness, which to some extent restricts high-resolution visualization of thicker regions. As a result, most *in situ* cryo-EM studies have focused on parasites from the phylum Apicomplexa, such as *Plasmodium*, *Toxoplasma*, and *Cryptosporidium*. These studies, likely facilitated by the small size of these organisms, have predominantly investigated the cell invasion machinery in isolated parasites. Works involving infective stages interacting with host cells have only recently emerged (11, 13–21). In contrast, the use of cryo-EM for *in situ* studies of whole cells in larger organisms, such as *Giardia* and *Trichomonas*, remains rare. Experiments in these organisms have primarily been limited to the analysis of isolated proteins or structures within membrane-extracted cells (22–27).

In pathogenic trypanosomatids, various approaches have been employed to address the limitations posed by cell thickness. These include the use of anucleated *Trypanosoma brucei* mutants (28), combining cryo-EM with STEM mode (29, 30), and producing cryosections via cryoultramicrotomy or lamellae through milling with a focused ion beam microscope (Cryo-FIB SEM) (31–33). Despite these advances, most studies have primarily focused on thinner cellular regions, particularly the flagellum of *T. brucei* (32, 34–36). However, the structure of thicker regions and, more importantly, the physiological processes occurring in these areas remain largely unexplored.

In this review, we revisited the contributions of electron microscopy (EM) methods to parasite research, with a particular emphasis on acidocalcisomes, multifunctional organelles that play essential physiological roles in a wide variety of cells. Additionally, we explore how cryo-EM can uncover new aspects of protozoan biology, broadening our current understanding of their structure, function, and physiology.

## THE ACIDOCALCISOMES

Acidocalcisomes are organelles ranging between 100 and 600 nm in diameter, characterized by an acidic, electron-dense matrix composed of phosphate polymers (polyphosphates [PolyP]) bound to ions. They were initially described in various organisms, including bacteria, plants, and protists—such as trypanosomatids—where they were originally referred to as volutine granules (37–41). Later, this organelle was identified in yeast, where they were termed “polyphosphate bodies” due to the presence of polyphosphate chains (37, 42, 43). The definitive naming of acidocalcisomes came after the identification of proton and calcium pumps on their surface in *T. brucei* and *T. cruzi* (43–45). Acidocalcisomes have since been observed in other trypanosomatids (*Leishmania* spp., *Leptomonas* spp., *Phytomonas*, and various monogenetic parasites), as well as in diverse groups, such as Apicomplexa (*Toxoplasma gondii* and *Plasmodium* spp.), algae (*Chlamydomonas reinhardtii*), fungi (*Dictyostelium discoideum*), insects, echinoderms, birds, and mammals, including human platelets, indicating their evolutionary conservation (43, 46–53).

In trypanosomatids, acidocalcisome membranes contain proteins that regulate the pH and ion composition of the matrix, including proton pumps, such as the vacuolar H^+^-pyrophosphatase (V-H^+^-PPase) (54), vacuolar H^+^-ATPase (V-H^+^-ATPase), and calcium ATPase (Ca^2+^-ATPase) (45, 55). Other key components include the zinc transporter (ZnT) and the inositol 1,4,5-trisphosphate receptor (IP3-R) (56–60). Additionally, a polyphosphate kinase (VTC) (61, 62), involved in PolyP synthesis and translocation, and the water channel aquaporin 1 (AQP1) (63) have also been identified on the membrane of acidocalcisomes.

Acidocalcisomes play crucial roles in osmoregulation, pH control, cellular homeostasis, calcium signaling, and cell bioenergetics (64). In trypanosomatids, the pH of acidocalcisomes adjusts in response to H^+^ concentrations in the cytoplasm, mitochondrial matrix, and other acidic compartments, including the acidocalcisomes themselves (65). A decrease in the activity of the proton pumps within acidocalcisomes leads to reduced organelle acidification and a corresponding drop in cytoplasmic pH (66).

Additionally, acidocalcisomes serve as the primary reservoir for intracellular Ca^2+^. Calcium uptake and release, mediated by Ca^2+^-ATPase and inositol 1,4,5-trisphosphate receptor (IP3-R), respectively, position this organelle as central to calcium signaling. These processes directly influence mitochondrial activity, cellular bioenergetics, and parasite infectivity (59, 67–69).

The electron-dense, acidified content of acidocalcisomes, rich in Ca^2+^ and phosphate chains (polyP), is a common feature across many organisms, although variations exist in the ion composition of the matrix. For instance, in *C. reinhardtii*, calcium, magnesium, and zinc ions have been identified in acidocalcisomes (47, 70), whereas in human platelets, only calcium and potassium ions have been found (46).

In trypanosomatids, the acidocalcisome matrix contains sodium, magnesium, potassium, calcium, iron, zinc, and phosphate polymers (53, 71–74). However, differences in the number, volume, and diameter of acidocalcisomes, as well as the concentrations of ions within their matrix, have been registered across species (73).

## ACIDOCALCISOME STRUCTURE AND COMPOSITION UNVEILED THROUGH DIFFERENT EM PREPARATION AND IMAGING TECHNIQUES

### Chemical fixation: advantages and limitations

Fixation is the initial and most critical step in the preparation of biological samples for electron microscopy. Its primary purpose is to halt biological activity, preserving the structural organization and three-dimensional integrity of the sample with minimal alteration (75). This can be achieved either chemically, using fixation reagents, or mechanically, through cryofixation, using various methods.

Chemical fixation fundamentally involves the immobilization of cellular components through the formation of cross-links between and within molecules (76, 77). The primary fixatives commonly used for routine electron microscopy are aldehyde-based fixatives, such as formaldehyde and glutaraldehyde, which provide enhanced preservation of cellular components when combined (78). Following aldehyde fixation, osmium tetroxide is typically used to stabilize lipids and unsaturated molecules, providing improved contrast for electron microscopy analysis (79).

Despite the mechanisms of fixative reagents in immobilizing cellular structures (80, 81), the integrity of the cell membranes can be compromised during the fixation process, allowing for the diffusion of molecules that may lead to significant artifacts. Fixation with glutaraldehyde, osmium tetroxide, or a combination of both completely abolishes the selective permeability of membranes, directly interfering with the osmotic properties of cell membranes. This disruption renders them permeable to osmolytes and leads to the loss of essential ions, such as potassium and magnesium, along with ATP (82). Additionally, fixation can cause the redistribution of proteins and lipids within the sample, stimulus of membrane vesiculation or blebs, leading to cell shrinkage or swelling (80, 82–85).

Considering the ionic and acidic nature of acidocalcisomes, studying their ultrastructure using chemical fixation presents a significant challenge. They often appear as empty vacuoles containing remnant or degraded electron-dense material when fixative reagents are applied (64, 72–74, 86). While alternative fixation solutions can mitigate these artifacts (73), subsequent steps in conventional TEM sample preparation—such as dehydration, resin embedding, and ultramicrotomy—can introduce additional structural alterations (87–89).

### Energy-filtered transmission electron microscopy of whole cells

**Fig 1:**
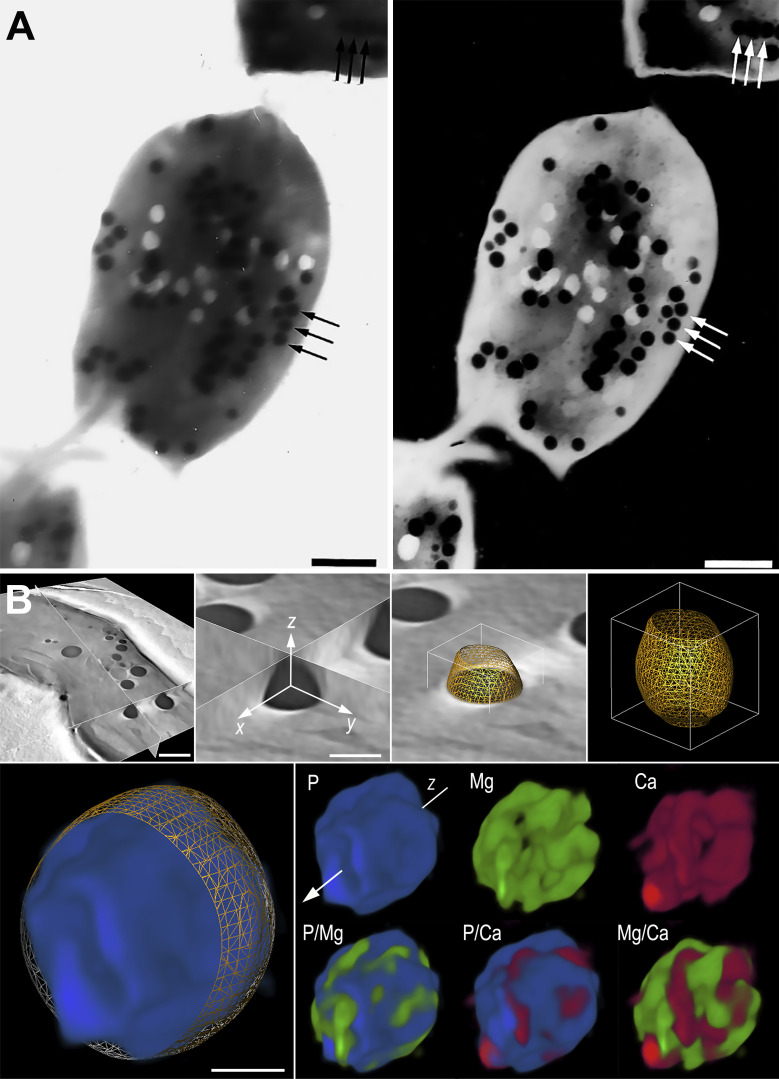
Visualization of acidocalcisomes in *T. cruzi* using different methodologies. (A) TEM image of an unfixed whole amastigote observed in conventional bright-field mode (left) and in electron spectroscopic imaging using contrast tuning (right), where the inelastic imaging mode provides optimal visualization of the acidocalcisomes, which are arranged in alignment (arrows). Scale bars: 700 nm. (B) Virtual section from an electron tomogram showing acidocalcisomes with 3D elemental mapping models, revealing the presence of ion nanodomains within the organelles. Scale bars = 500, 300, and 100 nm, from top left to bottom. (Panel A was reproduced from reference 72 with permission; panel B was reproduced from reference 91 with permission.)

Due to the high density of their core matrix, acidocalcisomes are relatively easy to visualize *in situ* using transmission electron microscopy (TEM) without requiring prior treatment. This can be achieved by adhering cells to grids coated with a continuous layer of carbon or formvar, blotting them with filter paper, and directly visualizing them at the TEM (72, 90). High-resolution imaging of whole cells is fundamentally limited by sample thickness, which induces chromatic aberration. Significant improvements in resolution and contrast to visualize intracellular structures in whole cells can be achieved with the use of an energy-filtering TEM, which allows for the selection of electrons within specific energy loss ranges (contrast tuning), reducing chromatic aberration (72–74) (Fig. 1A).

### Understanding acidocalcisome structure through cryofixation techniques

Cryofixation has been widely applied in the study of acidocalcisomes, with minor methodological variations tailored to specific objectives, whether ultrastructural, elemental analysis, or both. Each approach has significantly contributed to our understanding of this organelle; however, they also present certain limitations. Addressing both the advantages and limitations of these methods may provide valuable insights to guide future advancements in the field.

The principle of any cryofixation technique is the freezing of intracellular water from a liquid to a solid state at rapid or ultrarapid rates (around −73°C to 10^−4^ s) avoiding ice crystal formation—a process known as vitrification (92–95). This rapid immobilization of water halts physiological processes within milliseconds, eliminating the need for chemical fixatives. Consequently, cryofixation preserves cellular structures in a near-native, hydrated state, potentially achieving sub-nanometer resolution in ultrastructural analysis (96–98). Vitrification can be achieved through several methods, including the widely used plunge freezing at high velocity in atmospheric conditions or high-pressure freezing techniques, which enable vitrification of thicker samples (99, 100).

Plunge freezing involves quickly submerging a sample-carrying grid or metal support into a liquid cryogen, which has been pre-cooled with liquid nitrogen. This technique, pioneered by Dubochet et al. in the 1980s (92, 93, 101), can be performed using manual plungers or modern semi-automated systems. Commonly used alkanes include freon, propane, or ethane due to their high thermal conductivity and boiling points, ensuring rapid heat extraction (102–104). Additionally, variables, such as ambient humidity and blotting parameters used during plunging, are essential for effective vitrification (94, 103).

High-pressure freezing (HPF) is a technique that applies approximately 2,100 bar of pressure, allowing samples to reach liquid nitrogen temperatures (~−196°C) within about 20 ms (99). The high pressure also prevents water expansion, which reduces ice nucleation and slows crystal growth, optimizing vitrification and preserving cellular structures without ice crystal formation (99, 100, 105, 106). Due to the low thermal conductivity of water, which limits heat transfer from the sample surface to its center, HPF is suitable for specimens up to 600 µm thick, including eukaryotic cells, tissues, organoids, and even whole organisms (99, 100, 104).

Once a biological sample is cryofixed, it undergoes TEM analyses. Most eukaryotic cells must be thinned to reach an optimal thickness to achieve a high signal-to-noise ratio (SNR), minimizing image blurring and ensuring the acquisition of high-resolution data (107). Among the methods used to reach deeper cell regions, conventional electron microscopy of vitreous sections (CEMOVIS) was employed for the analytical investigation of acidocalcisomes in trypanosomatids (90). In this method, a bulk vitrified sample is mounted onto a metal holder and sectioned into slices 100–200 nm thick under cryogenic conditions (108–110). Unlike ultramicrotomy performed at room temperature, these sections are typically collected on grids under dry conditions to avoid the use of floating liquids, which could alter the ultrastructure or the distribution of diffusible substances within the sample (108, 111). This method inherently introduces some artifacts due to its nature: (i) compression and cracks primarily resulting from localized stress as the sample passes through the knife edge and (ii) dry transfer of sections to the grid followed by mechanical pressure to achieve adhesion. Both steps can produce morphological deformations in the sample, which may limit the potential for high-resolution studies (111, 112).

Freeze-drying (FD) is one of the possible dehydration processes after cryofixation. It removes water from biological samples by sublimation under low temperature and pressure, preserving structural integrity, improving stability, and enhancing contrast for electron microscopy (111, 113, 114). Freeze-dried (FD) cryosections are more stable when interacting with the electron beam, resulting in less damage and lower mass loss compared with hydrated cryosections (115). Thus, FD sections are widely used in microanalyses, such as electron energy loss spectroscopy (EELS) or energy-dispersive X-ray spectroscopy (EDS) (topics discussed in detail in the following section). In the case of hydrated sections, these are usually thicker (approximately 1 µm) to withstand the dose required for element detection without complete structural degradation (111, 115).

Despite these advantages, water extraction can alter biomolecular configurations, displace ions (particularly in compartments with high water content), and cause shrinkage of cellular structures (111, 113, 116). Additionally, the FD technique is primarily suitable for ultrastructural analyses at intermediate magnifications and has been so far insufficient for molecular structure studies that demand high resolution (111, 116).

Using CEMOVIS prior to elemental analysis, Scott et al. (90) demonstrated well-preserved epimastigote cells of *T. cruzi*, containing what they described as “spherical vacuoles of high mass density surrounded by a single membrane”—in other words, the acidocalcisomes. All observed acidocalcisomes showed a well-preserved core content with a dense matrix (90). In at least two acidocalcisomes, the membrane appeared separated from the core, a feature that could be interpreted as an artifact but allowed a clearer visualization of the membrane. Similar methodologies have continued to be applied in further analytical studies (discussed in the next section), consistently demonstrating the integrity and high density of the acidocalcisome matrix. However, the membrane and its interactions with other cytoplasmic structures were not always visible (72, 74).

An alternative methodology for dehydrating biological samples, which offers improved ultrastructural preservation, is freeze substitution (FS) following cryofixation, typically via HPF. The FS process replaces cellular water with chemical fixatives dissolved in an organic solvent at low temperatures. Samples are immersed in an FS medium, composed of ethanol, acetone, or methanol, combined with chemical fixatives that remain inactive until specific temperatures are reached (113, 117). The temperature of the sample is gradually increased to allow uniform stabilization of cellular structures throughout the entire cell volume via chemical fixation, thus reducing the likelihood of osmotic issues. This temperature progression can be automated using commercially available devices or manually controlled (113, 117, 118). After freeze substitution, samples undergo resin infiltration and embedding, as in conventional electron microscopy preparation.

Cryofixation combined with FS has significantly enhanced our ability to visualize structural details in a state closer to their native state (119–121), including entirely preserved acidocalcisomes in *T. cruzi*, revealing both the highly dense core and the surrounding membrane with its phospholipid bilayer (72). Over the past 20 years, this method has been increasingly applied, particularly in conjunction with volume microscopy techniques in the field of protozoology (121–127). Unlike single ultrathin or cryoultrathin sections that capture only two-dimensional images, these techniques allow for the detailed visualization of complex cellular and subcellular structures within a given volume, offering a better comprehension of spatial relationships (128–130).

Electron tomography effectively overcomes the resolution limitations inherent in other techniques by using virtual sectioning in semi-ultrathin sections (~200 nm) during tomogram reconstruction. This process enables the automated acquisition of high-resolution images with excellent lateral and *z*-axis resolution (128, 131). The ability to perform high-resolution volumetric imaging and reconstruction has opened up new opportunities to observe rarely visualized dynamic cellular events, such as acidocalcisome interactions with the contractile vacuole complex via membrane fusion (124, 132). This observation supports the role of acidocalcisomes in the osmoregulatory mechanism of *T. cruzi*, facilitating the transfer of ions and polyphosphates to the vacuole lumen and thereby increasing osmotic pressure within this organelle (63, 64, 132).

Even in ET of FS cells, acidocalcisomes displayed an empty matrix, with residual electron-dense material localized at the inner membrane surface (124). This phenomenon may have resulted from contact of the sections with water during the ultramicrotomy step prior to tomogram acquisition. This issue particularly affects diffusible cellular components, such as the ion-rich core of acidocalcisomes, which can be extracted from the section in regions exposed to water (113).

### Advances in the elemental detection methods

Determination of ion content, distribution, and concentration within cells is essential for understanding various cellular processes. Among the techniques used for this purpose, electron microscopy-based methods, particularly electron energy-loss spectroscopy (EELS) and related methods, and energy-dispersive X-ray spectroscopy (EDS), have been proven to be the most effective methods for high-resolution, subcellular *in situ* visualization of ion distribution within cells and organelles, such as acidocalcisomes.

Electron energy-loss spectroscopy (EELS) is carried out using a transmission electron microscope or a scanning transmission electron microscope (STEM) equipped with an energy filter (energy-filtering transmission electron microscope [EFTEM]). In both cases, the technique measures the electron energy loss after the interaction of the primary electron beam with the sample and can be applied on thin films of plunge-frozen samples using a cryoelectron microscope (133). EELS can provide information more than just elemental composition, such as thickness, quantitative information, bound elements, and oxidation states, and be combined with electron tomography to 3D elemental mapping of ions with high sensitivity at subnanometer resolution, which is particularly of great interest in the biological field (134, 135). Despite its superior sensitivity and spatial resolution for detecting elements at very low concentrations (90, 134, 136), its application to biological samples remains challenging due to their susceptibility to beam damage. Nevertheless, numerous studies have employed EELS methods to characterize acidocalcisomes in different protozoan parasites. Most of these studies have focused on elemental composition analysis to understand physiological aspects (137, 138) or ultrastructural analysis with contrast tuning (72, 74, 139–141).

Electron probe X-ray microanalysis (EPXMA), also known as energy-dispersive spectroscopy (EDS), has been used for decades as a powerful method to study the composition, distribution, and quantification of chemical elements in various materials and biological samples (142–146). This technique can detect simultaneously nearly all elements present in the sample with the advantage of correlating composition with ultrastructural details. The principle of X-ray microanalysis is based on the generation of X rays when the electron beam interacts with the atoms of the sample. X rays are collected by a detector positioned close to the sample, and a system classifies them by energy to produce a spectrum. The resulting histogram provides qualitative and quantitative information about the elemental composition of the sample (142). X-ray microanalysis can be performed using either scanning or transmission electron microscopy (S/TEM), enabling the analysis of samples ranging from thin sections to whole cells. Since diffusible elements are often lost during conventional preparation methods, the gold standard for X-ray microanalysis includes cryofixation techniques, such as plunge freezing, cryosectioning for thick samples, and freeze-drying as previously discussed (142, 147). These methodologies have been key tools for determining the chemical composition and, consequently, a better understanding about physiological processes that take place in acidocalcisomes of different organisms (43, 147, 148).

Advances in multi-detector systems, or array configurations, integrated with STEM have significantly enhanced detection sensitivity, enabling faster and more precise elemental mapping (149, 150). Recently, our group demonstrated the presence of ion nanodomains within acidocalcisomes of whole *T. cruzi* cells using a workflow that combined cryofixation with elemental mapping on a STEM microscope equipped with a system of four integrated EDS detectors and electron tomography for 3D elemental mapping. This study revealed a self-excluding pattern of cationic elements, such as magnesium and calcium (Fig. 1E) (91). These findings highlighted distinct features of the acidocalcisome core, providing the first evidence for a mechanism of formation of biomolecular condensates within an organelle, thus contributing to our understanding of the physiology of this organelle that may expand to other cell types. Moreover, ongoing advancements in X-ray mapping techniques continue to improve resolution and sensitivity, enabling exploration of three-dimensional elemental distributions (151). When integrated with cryo-EM, these innovations may significantly expand the scope of analytical studies on soft materials, with potential impact in various biological samples, including molecules.

## THE ACIDOCALCISOME IN THE AGE OF THE RESOLUTION REVOLUTION

The “resolution revolution” of biological sample imaging has been fueled by significant advancements in cryo-transmission electron microscopy (TEM), particularly through the development of microscopes operating at 300 kV and equipped with field emission guns (FEGs). These microscopes generate highly coherent electron beams with increased penetration power, enabling high-resolution imaging (152). Additionally, the introduction of direct electron detectors (DEDs) has transformed cryo-EM by enabling individual electron event detection and dose distribution across multiple frames. This technology allows motion correction, enhances imaging flexibility, and significantly improves image quality (153, 154).

To counteract the inherent low signal-to-noise ratio typically found when imaging biological samples—due to the use of low-dose imaging techniques—energy filters have been employed to remove electrons that have suffered energy loss and contribute to chromatic aberration. In this so-called zero-loss imaging, only unscattered and elastically scattered electrons that come through the objective aperture are retained, improving image contrast (155–157). Further contrast enhancement can be achieved by defocusing or with the use of phase plates (158, 159).

Advancements in cryo-focused ion beam scanning electron microscopy (cryo-FIB SEM) have significantly contributed to the improvement of resolution in biological cryo-EM. Cryo-lamellae preparation and lift-out approaches enable precise thinning and preparation of samples for high-resolution structural analysis, overcoming limitations of traditional methods like CEMOVIS (14, 31, 160, 161). By preserving structural integrity and minimizing artifacts, cryo-FIB SEM enhances sample quality and supports clearer imaging, making it an invaluable tool for cryo-ET (14, 160, 162). Additionally, combining cryo dual-beam microscopy with cryo-fluorescence microscopy allows precise localization of specific structures or molecules within complex samples. This integration is particularly useful for physiological studies, offering detailed contextual insights into biological systems (163–166).

Although there are no published cryo-EM studies fully dedicated to investigating the acidocalcisome yet, evidence suggests that these new technologies could be important tools to improve our understanding of this organelle and its physiological functions.

To investigate the impact of *T.brucei* flagellar movement on the parasite structure, Sun et al. (28) used cryo-ET to observe whole zoid cells (anucleate cells) displaying structural modifications in the flagellar attachment zone. Although not explicitly identified by the authors, numerous electron-dense granules were described, ranging from 100 to 350 nm in diameter, resembling the well-characterized acidocalcisome in trypanosomatids (28). Those electrodense granules were observed in close contact with the mitochondria. Similarly, the interaction between mitochondria and acidocalcisomes has been suggested in *Chlamydomonas*, *T. brucei*, and *T. cruzi*. Indirect evidence, primarily based on the proximity of these organelles in ultrathin sections and super-resolution fluorescence microscopy images, has been documented (59, 72, 167, 168). In addition to identifying contact sites between these organelles, studies in *T. brucei* have implicated the IP3 receptor (located on acidocalcisome membrane), the voltage-dependent anion channel (VDAC; on the outer mitochondrial membrane), and the mitochondrial calcium uniporter (MCU; on the inner mitochondrial membrane) as mediators of Ca²^+^ ion transfer from the acidocalcisome to the mitochondrion (168, 169). In *T. cruzi*, deletion of the receptor TcIP3R disrupted the parasite’s metabolism by decreasing mitochondrial Ca²^+^ uptake, which inhibited O₂ consumption and oxidative phosphorylation (59).

Although calcium transfer to mitochondria in eukaryotes is well-established, research on the mechanism, quantity, and bioavailability of calcium within the mitochondria remains limited mainly due to the challenges in preserving ions for imaging and detection, as previously discussed. However, this scenario seems to be changing with the recent cryo-ET improvements. A study on osteoblasts subjected to high-pressure freezing and freeze substitution, followed by EDX, identified electron-dense granules in the mitochondrial matrix composed of calcium phosphate (170). Later, a similar result was observed in fibroblasts through cryo-ET in scanning transmission electron microscopy (STEM) combined with EDX, showing amorphous and dense granules dispersed in the mitochondrial matrix also composed of calcium phosphate (30). Given the widespread presence of acidocalcisomes and acidocalcisome-like structures across diverse biological groups, these findings further reinforce the role of this organelle not only in the storage of essential ions but also in its interactions with organelles, such as mitochondria and its involvement in various physiological processes, including bioenergetics.

**Fig 2:**
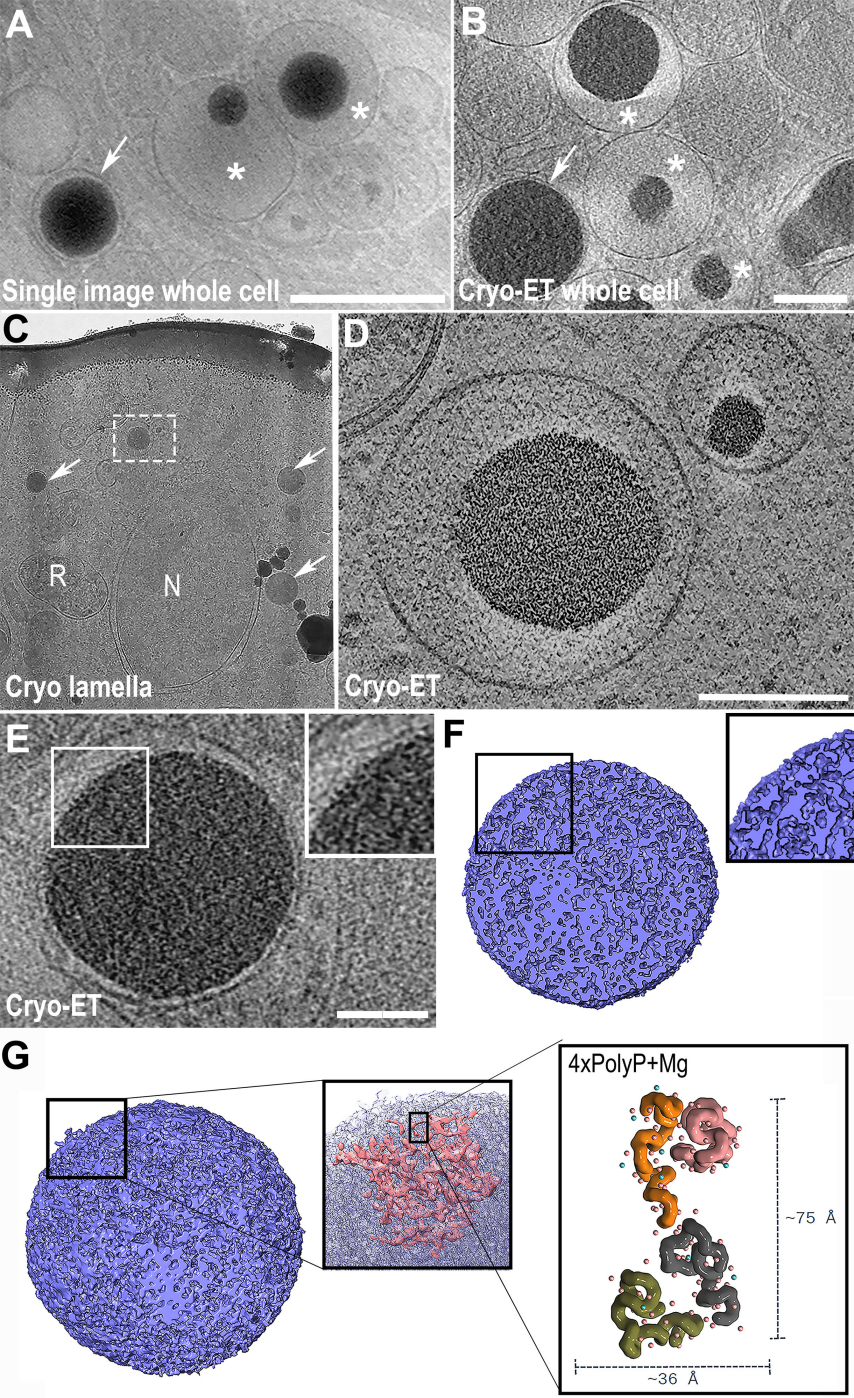
Visualization of acidocalcisomes in *T. cruzi* using cryo-EM and cryo-ET. (A and B) A single image and a virtual section from a whole cell tomogram, respectively. Both images show different patterns of acidocalcisome structure, the fully loaded matrix containing a well-preserved electrodense core (arrows) and the partially filled matrix with cores of different diameters (asterisks). In these images, the surrounding and intracellular interactions with the acidocalcisomes can be clearly observed. Scale bar = 500 and 200 nm, respectively. (C) TEM image of a ~200 nm lamella from an epimastigote form of *T. cruzi*. To minimize beam damage and acquire an overview for a search map, images of lamellae are initially acquired at low magnification, which reduces the signal-to-noise ratio (SNR). The nucleus (N), reservosomes (R), and acidocalcisomes are visible, with their matrix either fully (arrows) or partially filled (dotted square). (D) Cryo-ET virtual section of the acidocalcisomes from the dotted square region in panel A, showing both the membranes and electrodense cores with high resolution. Scale bar = 200 nm. (E) Cryo-ET of whole cell virtual section, displaying fine details of the acidocalcisome matrix texture (inset), potentially representing the *in situ* observation of aggregates of polyphosphate molecules. Scale bar = 100 nm. (F) 3D model based on the threshold of the acidocalcisome matrix shown in panel E. (G) The final 3D model reveals highly interconnected structures, which, when closely observed, resemble the conformation and size of polyphosphate chains in the presence of ions such as Mg²^+^. Conformation models obtained from molecular dynamics based on four polyphosphate chains with 50 phosphates and magnesium over a 100 ns simulation are shown. (Panel A was reproduced from reference 91 with permission.)

Cryofixed whole cells of *T. cruzi* analyzed by single-image cryo-EM and cryo-ET revealed acidocalcisomes with varying electron-dense core patterns (91). Some displayed a fully filled electron-dense matrix, while others appeared only partially filled (Fig. 2A and B) (91). Similarly, partially filled acidocalcisomes were observed in *Chlamydomonas* using cryo-FIB-SEM lamella analyzed by cryo-ET (33). The use of cryo-FIB-SEM lamella on sample preparation minimizes the artifacts commonly associated with traditional sectioning techniques (Fig. 2C), allowing for high-resolution visualization of ion-rich subcellular compartments in their hydrated state (Fig. 2D). This approach opens new paths for studying the ultrastructure and function of acidocalcisomes under near-native conditions. Preliminary data from cryo-ET analysis of acidocalcisomes revealed a distinct texture in the matrix, characterized by “fiber-like” regions (Fig. 2E). When modeled, the fibers exhibit dimensions very similar to those expected for polyPs, likely corresponding to the visualization of aggregates of polyphosphate molecules (Fig. 2F). Molecular fitting analysis, considering polyphosphate dimensions and interactions with divalent ions (91), aligns well with these observations and the measurements obtained from cryo-ET models (Fig. 2G). All evidence points to the possibility that we are observing polyphosphate aggregates *in situ* (Movie S1).

The variability in acidocalcisome matrix patterns observed in hydrated cells at high resolution underscores the need to reconsider the prevalent assumption that acidocalcisomes, in their native state, consistently feature a fully electron-dense matrix, and that any deviation from this morphology is merely a technical artifact. Moving beyond this assumption opens the possibility that a partially filled matrix could represent a native physiological state, potentially indicative of ion mobilization for maintaining homeostasis or as a stress response. Previous studies have already indicated substantial ion mobilization and ultrastructural dynamics within this organelle. In *Leishmania amazonensis*, a polymorphic acidocalcisome core was documented, showing dynamic changes in elemental composition and concentration in response to varying extracellular nutritional conditions, suggesting a high degree of ion storage shifts and mobility that result in ultrastructural changes (74). Similar ultrastructural diversity in the acidocalcisome matrix has also been reported in *Phytomonas françai* (53).

Thus, if the observed matrix patterns in acidocalcisomes represent distinct physiological stages of this organelle, could intermediate stages of ion mobilization be captured using cryo-EM? Furthermore, is it possible to visualize the nanodomains described within the acidocalcisome core with the latest advancements in cryomicroscopy? Recent findings suggest a promising future in this direction (Fig. 2).

A final question arising from this discussion is whether the high-resolution capabilities of cryo-EM could be effectively combined with elemental detection methods. The potential of such a combination could offer detailed ultrastructural information on acidocalcisomes in hydrated conditions, along with a comprehensive ion profile and mobilization—not only within the acidocalcisome matrix but also in interacting organelles, such as mitochondria and contractile vacuole. Capturing these dynamic processes in a near-native state could be instrumental in understanding the role of acidocalcisomes in parasite physiology

## CONCLUSION AND FUTURE PERSPECTIVES

The use of cryo-EM to reveal new structural aspects of pathogens at the molecular level is expected to continue expanding in the coming years, provided that technological development investments remain strong. Just as correlative light electron microscopy (CLEM) has evolved to extend cryo-EM beyond mere morphological characterization, the integration of cryoelectron microscopy with elemental ion detection technologies holds significant potential to push existing boundaries. Many of the tools required to make this combination a reality are already available. On one hand, transmission electron microscopes equipped with sensitive multi-detector systems and capable of running electron tomograms, including cryo-ET, are accessible, though improvements in the column design could help address current limitations in angular range and the use of cryo-holders. On the other hand, to enhance contrast, most 300 kV TEMs are now equipped with energy filters. Despite being the most widely used instrument for high-end cryo-EM, the current generation equipment has significant limitations in fully leveraging the electron energy loss spectrum, featuring slit aperture controls ranging from 1 to 500 eV, limiting its functionality primarily to zero-loss imaging. Expanding support for elemental detection through EELS and optimized contrast (tuning) for whole-cell analyses, particularly integrating the latest laser phase plate technologies, holds tremendous potential for advancing the field.

In addition to their role in cell physiology, the understanding of Ca²^+^ storage nanostructures and the modulation of their binding to the support matrix, such as polyphosphate in acidocalcisomes, modulated by pH variation, is also in line with technological research on calcium-based batteries (171). The structural investigation of acidocalcisomes, in this sense, may also suggest polymers similar to polyphosphates as efficient matrices for the storage and release of divalent ions for these batteries, such as Ca^2+^ and Mg^2+^, which are much more abundant than the monovalent lithium.

Regardless of the specific advancements in cryo-EM future, its contribution to biological sciences and the study of infectious diseases remains undeniable. Expanding the application of cryo-EM has the potential to deepen our understanding of protozoan parasite ultrastructure and physiology, offering new perspectives for therapeutic development.

## SUPPLEMENTAL MATERIAL

Legend mbio.01662-24-s0001.docx Supplemental movie legend.

Movie S1 mbio.01662-24-s0002.mp4 *In situ* visualization of an acidocalcisome by cryo-ET.

ASM does not own the copyrights to Supplemental Material that may be linked to, or accessed through, an article. The authors have granted ASM a non-exclusive, world-wide license to publish the Supplemental Material files. Please contact the corresponding author directly for reuse.
