# Supplementary material for: New dimensions in acidocalcisome research: the potential of cryo-EM to uncover novel aspects of protozoan parasite physiology
Source: mBio. 2025 Apr 8;16(5):e01662-24. doi: 10.1128/mbio.01662-24 (PMC12077218; doi:10.1128/mbio.01662-24)
Supplement: Legend — Supplemental movie legend. [file mbio.01662-24-s0001.docx]

**Supplemental Material**

**Movie S1. In situ visualization of an acidocalcisome by Cryo-ET.** Cryo-electron tomogram reveals acidocalcisome matrix at high resolution, displaying a distinct texture pattern that likely represents aggregates of polyphosphate molecules in situ. The segmented 3D model of the acidocalcisome matrix (blue) shows highly interconnected structures (partially displayed in pink). These structures resemble the size and conformation of polyphosphate chains in the presence of magnesium ions, as predicted by molecular dynamics simulation analyses (based on four polyphosphate chains with 50 phosphates each, surrounded by magnesium over 100 ns).
